# Gender-independent efficacy of mesenchymal stem cell therapy in sex hormone-deficient bone loss via immunosuppression and resident stem cell recovery

**DOI:** 10.1038/s12276-018-0192-0

**Published:** 2018-12-17

**Authors:** Bing-Dong Sui, Ji Chen, Xin-Yi Zhang, Tao He, Pan Zhao, Chen-Xi Zheng, Meng Li, Cheng-Hu Hu, Yan Jin

**Affiliations:** 1 State Key Laboratory of Military Stomatology, Center for Tissue Engineering, Fourth Military Medical University, Xi’an, Shaanxi 710032 China; 2 Research and Development Center for Tissue Engineering, Fourth Military Medical University, Xi’an, Shaanxi 710032 China; 3 Xi’an Institute of Tissue Engineering and Regenerative Medicine, Xi’an, Shaanxi 710032 China

## Abstract

Osteoporosis develops with high prevalence in both postmenopausal women and hypogonadal men. Osteoporosis results in significant morbidity, but no cure has been established. Mesenchymal stem cells (MSCs) critically contribute to bone homeostasis and possess potent immunomodulatory/anti-inflammatory capability. Here, we investigated the therapeutic efficacy of using an infusion of MSCs to treat sex hormone-deficient bone loss and its underlying mechanisms. In particular, we compared the impacts of MSC cytotherapy in the two genders with the aim of examining potential gender differences. Using the gonadectomy (GNX) model, we confirmed that the osteoporotic phenotypes were substantially consistent between female and male mice. Importantly, systemic MSC transplantation (MSCT) not only rescued trabecular bone loss in GNX mice but also restored cortical bone mass and bone quality. Unexpectedly, no differences were detected between the genders. Furthermore, MSCT demonstrated an equal efficiency in rectifying the bone remodeling balance in both genders of GNX animals, as proven by the comparable recovery of bone formation and parallel normalization of bone resorption. Mechanistically, using green fluorescent protein (GFP)-based cell-tracing, we demonstrated rapid engraftment but poor inhabitation of donor MSCs in the GNX recipient bone marrow of each gender. Alternatively, MSCT uniformly reduced the CD3^+^T-cell population and suppressed the serum levels of inflammatory cytokines in reversing female and male GNX osteoporosis, which was attributed to the ability of the MSC to induce T-cell apoptosis. Immunosuppression in the microenvironment eventually led to functional recovery of endogenous MSCs, which resulted in restored osteogenesis and normalized behavior to modulate osteoclastogenesis. Collectively, these data revealed recipient sexually monomorphic responses to MSC therapy in gonadal steroid deficiency-induced osteoporosis via immunosuppression/anti-inflammation and resident stem cell recovery.

## Osteoporosis: Stem cell therapy success for both sexes

Stem cell therapy shows promise in reversing the bone loss caused by sex hormone deficiency in male and female mice. Adult stem cells, known as mesenchymal stem cells (MSCs), have been shown to facilitate bone healing when administered into the caudal vein of mice. Yan Jin and Cheng-Hu Hu at Fourth Military Medical University in Xi’an, China, and co-workers investigated the efficacy of MSC-based therapy in treating osteoporosis triggered by the loss of sex hormones. They generated mouse models with sex hormone deficiencies, and showed that the resulting osteoporosis seen was remarkably similar in males and females. MSC transplantation resulted in the restoration of bone formation and bone resorption processes in both sexes. The transplanted MSCs also suppressed the inflammation associated with osteoporosis, allowing resident MSC populations in the bone marrow to recover bone damage.

## Introduction

Extensive bone loss is a common problem in postmenopausal women and hypogonadal men that can lead to significant morbidity and mortality, but the cure remains as an unfulfilled medical challenge^1–3^. In the most recent decade, mesenchymal stem cells (MSCs) have revealed great promise to jumpstart and facilitate bone healing^4–7^. In particular, systemic MSC transplantation (MSCT) has shown a remarkable efficacy in preventing and treating estrogen deficiency-induced bone loss^8–12^. However, in osteoporosis triggered by male hypogonadism, the effects of MSCT are still not understood. Actually, gender homogeneity and heterogeneity of the skeleton have long been described or debated^13^. The complicated issues make it difficult to predict whether MSCT exerts gender-specific anti-osteoporotic impacts or not. Therefore, evaluating the therapeutic outcomes of MSCT in gonadal steroid deficiency-induced osteoporosis of both genders is of interest, and this information would help to establish a gender-specific or gender-independent translational remedy.

Current understanding of the mechanisms underlying MSC therapy is that either the transplanted MSCs home to the targeted tissues where they exert direct functions^10,14,15^ or they modulate the systemic/local microenvironment without successful engraftment^8,9,16^. In cytotherapy for osteoporosis, we have previously shown that systemically infused MSCs can engraft in recipient bone marrow to re-establish deficient osteoblastogenesis under certain conditions^14^. On the other hand, systemic delivery of MSCs exerts strong immunosuppressive effects in autoimmune diseases and the related bone loss^16,17^. For the cellular basis of bone biology, the functional importance of resident MSCs in differentiating into osteoblasts and modulating osteoclastogenesis are gradually recognized. Disorders in this process may serve as critical features of the pathogenesis that underlies the imbalanced bone remodeling process^5,18,19^, particularly under estrogen/androgen deficiency^20,21^. However, it remains unclear whether homing or microenvironmental modulation mediates the potential efficacy of MSC therapy and whether the endogenous stem cell function is restored by allogeneic MSC transplantation.

In this study, we aimed to investigate the effects of MSC therapy on osteoporosis in gonadal steroid deficiency and to compare males with females to examine potential gender differences. Furthermore, we extended our research to examine the therapeutic mechanisms of allogeneic MSCs. In a series of in vivo and ex vivo experiments, our data collectively uncovered recipient sexually monomorphic responses to MSC therapy in gonadal steroid deficiency-induced osteoporosis via immunosuppression/anti-inflammation and resident stem cell recovery, which shed light on the gender-independent clinical utility.

## Materials and methods

### Animals

All experiments were approved by Fourth Military Medical University and were performed following the Guidelines of the Intramural Animal Use and Care Committee of Fourth Military Medical University. Twelve-week-old female and male wild-type (WT) C57BL/6 mice and green fluorescent protein (GFP)^+/+^ transgenic mice with a C57BL/6 background (all from the Laboratory Animal Center, Fourth Military Medical University, Xi’an, Shaanxi, China) were used. Mice were provided food and water ad libitum. All of the group allocations and experimental designs described below are shown in Supplementary Figure 1.

### Culture of mouse MSCs

Equal quantities of female and male mice were used as donors of MSCs. The MSCs and GFP-labeled MSCs (MSCs^GFP^) from GFP^+/+^ donor mice used in this study were isolated from mouse bone marrow as previously described^8,20,22^. Briefly, mouse bone marrow cells from the femora and tibias were seeded into culture dishes, incubated overnight, and rinsed with phosphate-buffered saline (PBS) to remove the non-adherent cells. The adherent cells were cultured with alpha-minimum essential medium (α-MEM) supplemented with 20% fetal bovine serum (FBS), 2 mM L-glutamine, 100 U/ml penicillin, and 100 g/ml streptomycin (all from Invitrogen, Carlsbad, CA, USA) at 37 ℃ in a humidified atmosphere with 5% CO_2_. The media were changed every 2 days during the growth of the MSC colonies. In some cases, the colonies were stained with crystal violet (Sigma-Aldrich, St. Louis, MO, USA) for observation after fixation with 4% paraformaldehyde (Sigma-Aldrich, St. Louis, MO, USA)^20^. After reaching confluence, other MSC colonies were digested with 0.25% trypsin (MP Biomedicals, Santa Ana, CA, USA) and were applied for either transplantation or verification by multilineage differentiation and surface marker expression^14,20,23^.

For surface marker profiling, after digestion, primary MSCs were suspended in PBS supplemented with 3% FBS at 1 × 10^6^ cells/ml. Aliquots of 2 × 10^5^ cells/tube were stained with 1 μl of FITC-conjugated anti-mouse CD11b antibody, 1 μl of PE-conjugated anti-mouse CD29 antibody, 1 μl of PE-conjugated anti-mouse CD34 antibody, 1 μl of PE-conjugated anti-mouse CD45 antibody, 1 μl of PE-conjugated anti-mouse vascular cell adhesion molecule 1 (VCAM1, also known as CD106) antibody, or 1 μl of FITC-conjugated anti-mouse stem cell antigen 1 (Sca1) antibody (all from Abcam, Cambridge, UK)^8,14^. After washing, the cells were analyzed using a flow cytometer (FACSAria; BD, Franklin Lake, NJ, USA), and the percentages of positively stained cells were determined using the FACSDiva Version 6.1.3 software (BD, Franklin Lake, NJ, USA).

### Experiment 1: Effects of MSCT on bone mass and quality in treating gonadectomy (GNX) mouse osteoporosis of both genders

WT mice were divided into three groups of each gender: the Sham group (*n* = 5/gender), the GNX group (*n* = 5/gender), and the MSCT group (*n* = 5/gender). For the model of osteoporosis, female mice were subjected to a bilateral Sham or ovariectomy (OVX) operation by the dorsal approach and male mice were subjected to a bilateral Sham or orchidectomy (ORX) operation by the abdominal approach, under general anesthesia, according to our previous studies^8,20^. The model was established in mice for 4 weeks subsequent to surgery, and mice then received the therapeutic interventions on the basis that the bone loss was expected to have developed after that duration^8,20^. For the Sham and GNX groups, 200 μl of PBS was infused intravenously. For the MSCT group, 1 × 10^6^ donor MSCs were suspended in 200 μl of PBS and injected via the caudal vein as previously established^8,14^. The transplanted MSCs were digested from primary colonies and used as a 1:1 mixture of MSCs derived from WT mice of both genders to prevent potential sex bias. Mice were maintained for another 4 weeks after treatments to allow recovery^8^ and were euthanized for sample collection from the femora at an age of 20 weeks. The left femora were prepared for bone mass evaluation, and the right femora were examined for bone quality.

For micro-computed tomographic (micro-CT) analysis of the bone mass, after sacrifice, the collected femoral samples were fixed overnight in 4% paraformaldehyde, and then 1-cm specimens were prepared that included the distal femoral metaphysis. The specimens were scanned at a resolution of 8 μm, using a voltage of 80 kV and a current of 80 μA in a desktop micro-CT system (eXplore Locus SP; GE Healthcare, Wauwatosa WI, USA)^24,25^. After image reconstruction, the regions of interest (ROI) for the analysis of trabecular bone were set from 0.3 to 0.8 mm away from the growth plates in the distal metaphysis^8,14^. The data, which included the parameters of bone volume over tissue volume (BV/TV), trabecular thickness (Tb.Th), trabecular number (Tb.N), and trabecular separation (Tb.Sp)^26^ were obtained using the Micview V2.1.2 software (GE Healthcare, Wauwatosa WI, USA). The cortical ROI was defined in the midshaft, from 3.3 to 3.8 mm away from the growth plates^14,25^. Cortical bone quantification was based on cortical thickness (Ct.Th) and cortical area (Ct.Ar) fraction over total area using the Micview V2.1.2 software (GE Healthcare, Wauwatosa WI, USA)^26^.

For the three-point bending mechanical test of bone quality, after sacrifice, the collected femoral samples were fixed overnight in 4% paraformaldehyde. The length was measured using calipers, and the midpoint was determined. The samples were then placed in a universal testing machine (AGS-10KN; Shimadzu, Kyoto, Japan) with the two lower supports at a distance of 15 mm apart. The load was applied to the midpoint at a displacement rate of 0.05 mm/s until failure. The displacement, the load and the load-deformation curve were recorded. Ultimate force was defined as the maximal load. Ultimate stress and Young’s modulus was calculated accordingly^14^.

### Experiment 2: Effects of MSCT on bone remodeling in treating GNX-induced osteoporosis in mice of both genders

WT mice were divided into three groups of each gender: the Sham group (*n* = 5/gender), the GNX group (*n* = 5/gender), and the MSCT group (*n* = 5/gender). Sham and GNX modeling, systemic administration of PBS and MSCs, and the experimental time period were the same as in Experiment 1. After 16 days, mice received an intraperitoneal injection of calcein, which was repeated 2 days prior to sacrifice, as previously described^8,14^ and in details below. Just before sacrifice, peripheral blood was collected for serum sampling. At sacrifice, the left femora were sampled for evaluation of the calcein-based bone formation, and the right femora were sampled to examine the bone resorption.

For bone formation examination by calcein double-labeling^8,14,20^, briefly, at 16 days and 2 days prior to sacrifice, mice received intraperitoneal injections of 20 mg/kg calcein (Sigma-Aldrich, St. Louis, MO, USA), which was dissolved at a concentration of 2 mg/ml in PBS supplemented with 1 mg/ml NaHCO_3_ (Sigma-Aldrich, St. Louis, MO, USA). After sacrifice, the collected femora were fixed in 80% ethanol, embedded in methyl methacrylate, and sagittally sectioned into 30-μm sections using a hard tissue slicing machine (SP1600; Leica, Solms, Germany) away from light. Both double-labeled and single-labeled cortical endosteum surfaces were evaluated using a fluorescence microscope (STP6000; Leica, Solms, Germany) at an excitation wavelength of 488 nm. Quantification was first performed using the parameters of mineral apposition rate (MAR) and the mineralized surface over bone surface (MS/BS), and the bone formation rate (BFR) was then calculated as MAR × MS/BS, as recommended^27^.

For bone resorption examination, tartrate-resistant acid phosphatase (TRAP) staining was employed^8,25^. After sacrifice, the collected femora were fixed overnight with 4% paraformaldehyde, decalcified with 10% ethylene diamine tetraacetic acid (EDTA), and embedded in paraffin. Serial 5 μm sagittal sections of the distal metaphyses at were prepared (RM2125; Leica, Solms, Germany) and were stained for TRAP activity using a commercial kit (387-1A; Sigma-Aldrich, St. Louis, MO, USA). Osteoclastic bone resorption quantification was determined using the parameters of the number of osteoclasts over the bone surface (N.Oc/BS) and osteoclast surface over bone surface (Oc.S/BS) using the ImageJ 1.47 software (National Institute of Health, Bethesda, MD, USA)^27^.

For the detection of serological markers, peripheral blood samples were collected from the retro-orbital venous plexus before sacrifice, and the serum was isolated by centrifugation at 3000 rpm for 10 min followed by 12000 rpm for 10 min at 4 ℃. Markers for osteoblastic bone formation (osteocalcin, OCN; procollagen 1 N-terminal peptide, P1NP), osteoclastogenesis (receptor activator of nuclear factor-κB ligand, RANKL; osteoprotegerin, OPG; TRAP-5b) and osteoclastic bone resorption (cross-linked C-telopeptide of type 1 collagen, CTX-1) were detected using enzyme-linked immunosorbent assay (ELISA) kits according to the manufacturers’ instructions (R&D Systems, Minneapolis, MN, USA)^8,9,25^.

### Experiment 3: Bone marrow tracing of infused MSCs in GNX mice of both genders

MSCs^GFP^ were used for transplantation in this experiment. WT mice that had previously been subjected to GNX were used as the recipients and were divided into two groups of each gender: the GNX + MSC^GFP^ – 24 h group (*n* = 3/gender) and the GNX + MSC^GFP^ – 4 w group (*n* = 3/gender). GNX modeling and systemic delivery of MSCs were performed according to the methods and time points described above. At 24 h and 4 w after the MSC^GFP^ infusion, mice were euthanized for femoral sample collection to evaluate the donor MSC engraftment in the recipient bone marrow, for immediate and long-term inhabitation, respectively^8,14^.

For immunofluorescent staining, after sacrifice at indicated time points, the collected femora were fixed overnight with 4% paraformaldehyde, decalcified with 10% EDTA, cryoprotected with 30% sucrose, embedded in the optimal cutting temperature (OCT) compound and snap-frozen. The samples were then sectioned into 15-μm sagittal sections (CM1950; Leica, Solms, Germany), blocked with 5% bovine serum antigen (BSA) for 1 h at room temperature, stained for GFP using a rabbit-anti-mouse primary antibody (Cell Signaling Technology, Boston, MA, USA) for 2 h at room temperature at 1:100, followed by a goat anti-rabbit-FITC secondary antibody (Cell Signaling Technology, Boston, MA, USA) at 1:200 for 30 min at room temperature, counterstained with Hoechst, and observed using a fluorescence microscope (DP70; Olympus, Tokyo, Japan). The percentages of GFP-positive cells in the total recipient bone marrow area were analyzed using ImageJ 1.47 software (National Institute of Health, Bethesda, MD, USA)^8,14,28^.

### Experiment 4: Effects of MSCT on the total T-cell population and inflammation during treatment of GNX-induced osteoporosis in mice of both genders

WT mice were divided into three groups of each gender: the Sham group (*n* = 8/gender), the GNX group (*n* = 8/gender), and the MSCT group (*n* = 8/gender). Sham and GNX modeling, systemic administration of PBS and MSCs, and the experimental time period were the same as those described for Experiment 1. Just before sacrifice, three mice of each group were randomly chosen for peripheral blood and bone marrow cell sampling for CD3^+^T cell determination, and blood was collected from the remaining mice for serum analyses. Mice were then euthanized.

For flow cytometric analysis for quantification of the total T-cell population, samples of peripheral blood were collected from the retro-orbital venous plexus before sacrifice, and samples of bone marrow were collected from femora. After being treated with ACK lysis buffer (Lonza, Basel, Switzerland) to remove the red blood cells, 1 × 10^6^ cells were incubated with 1 μl of FITC-labeled rabbit-anti-mouse CD3 antibody (Abcam, Cambridge, UK) for 40 min on ice. After washing twice with PBS, the percentages of total CD3^+^T cells in the peripheral blood mononuclear cells (PBMNCs) and the bone marrow mononuclear cells (BMMNCs) were determined using a flow cytometer (CytoFLEX; Beckman Coulter, Brea, CA, USA) equipped with the CXP 2.1 software (Beckman Coulter, Brea, CA, USA)^8,9,17^. For detection of serological markers, serum samples was isolated as described for Experiment 2, and markers of inflammation (Tumor necrosis factor-alpha, TNF-α; Interferon-gamma, IFN-γ) were determined using ELISA kits according to the manufacturers’ instructions (R&D Systems, Minneapolis, MN, USA)^8,9,25^.

For ex vivo T-cell culture and co-culture assays with MSCs, murine spleen cells derived from WT or GNX mice were collected and treated with ACK lysis buffer (Lonza, Basel, Switzerland) to remove the red blood cells as described previously^8^. The T cells were isolated and stimulated for 2 days with 3 μg/ml plate-bound anti-mouse CD3 antibody (eBioscience, San Diego, CA, USA) and 2 μg/mL soluble anti-mouse CD28 antibody (eBioscience, San Diego, CA, USA) in α-MEM supplemented with 20% FBS, 2 mM L-glutamine, 100 U/ml penicillin, and 100 g/ml streptomycin (all from Invitrogen, Carlsbad, CA, USA) at 37℃ in a humidified atmosphere with 5% CO_2_. Direct T-cell co-culture assays with MSCs were then performed^17^. Briefly, primary MSCs were seeded at 5 × 10^5^ cells/well in 6-well plates for 24 h. Stimulated T cells at 5 × 10^6^ cells/well were either cultured without MSCs (Control, Ctrl) or added to MSCs for 6 h. The media were collected and centrifuged at 12000 rpm for 10 min at 4 ℃, and the TNF-α and IFN-γ concentrations were detected using ELISA kits according to the manufacturers’ instructions (R&D Systems, Minneapolis, MN, USA). The T cells of the co-culture experiments were collected for the determination of T-cell apoptosis.

For apoptosis analysis, after the co-culture assays, T cells were harvested and evaluated using FITC-conjugated Annexin V and PI double staining according to the manufacturer’s instructions for the Annexin V Apoptosis Detection Kit I (BD Biosciences, San Jose, CA, USA). After incubation, cell apoptosis was evaluated using a flow cytometer (CytoFLEX; Beckman Coulter, USA) equipped with the CXP 2.1 software (Beckman Coulter, Brea, CA, USA). The percentages of early apoptotic (FITC^+^PI^−^) plus late apoptotic/necrotic (FITC^+^PI^+^) cells were expressed as apoptotic percentages, as stated^8^.

### Experiment 5: Effects of MSCT on resident MSC function in the treatment of GNX-induced osteoporosis in mice of both genders

WT mice were divided into three groups of each gender: the Sham group (*n* = 3/gender), the GNX group (*n* = 3/gender), and the MSCT group (*n* = 3/gender). Sham and GNX modeling, systemic administration of PBS and MSCs, and the experimental time period were as described for Experiment 1. Mice were euthanized, and resident MSCs were sampled. Isolated endogenous MSCs were then cultured for the assessment of osteogenesis using alkaline phosphatase (ALP) and alizarin red staining, and for the capability for the induction of osteoclastogenesis based on RANKL secretion and co-culture with osteoclasts.

For osteogenic differentiation of MSCs, after digestion, primary MSCs were seeded and induced in osteogenesis-inducing media containing 100 μg/ml ascorbic acid (MP Biomedicals, Santa Ana, CA, USA), 2 mM β-glycerophosphate (Sigma-Aldrich, St. Louis, MO, USA) and 10 nM dexamethasone. After induction for 7 days, ALP staining was performed using a commercial kit (Sigma-Aldrich, St. Louis, MO, USA) to determine the ALP activity associated with osteogenesis^25^. After induction for 14 days, alizarin red (Sigma-Aldrich, St. Louis, MO, USA) staining was performed to evaluate the mineralization^19,20^. For adipogenic differentiation of MSCs, after digestion, primary MSCs were seeded and induced in adipogenesis-inducing media containing 0.5 mM isobutylmethylxanthine (MP Biomedicals, Santa Ana, CA, USA), 0.5 mM dexamethasone and 60 mM indomethacin (MP Biomedicals, Santa Ana, CA, USA). After induction for 14 days, oil red O (Sigma-Aldrich, St. Louis, MO, USA) staining was performed to evaluate the lipid droplet formation^20,29^.

For the analysis of cytokine secretion, after digestion, primary MSCs were seeded and cultured for 3 days. The culture media were then collected, and the supernatants were harvested by centrifugation at 12,000 rpm for 10 min at 4 ℃^8^. The RANKL concentration was then determined using ELISA kits according to the manufacturers’ instructions (R&D Systems, Minneapolis, MN, USA). For induction of osteoclastogenesis, after digestion, primary MSCs were seeded at 2 × 10^5^ cells/well in 12-well plates. A new batch of total bone marrow cells was then isolated and incubated for 24 h, and the nonadherent cells were collected. The nonadherent cells were then seeded at 2 × 10^6^ cells/well onto the pre-seeded MSCs and cultured with 20 ng/ml macrophage colony-stimulating factor (M-CSF), 10 nM 1,25(OH)_2_-vitD3 (Sigma-Aldrich, St. Louis, MO, USA) and 1 µM prostaglandin E2 (PGE_2_) (Sigma-Aldrich, St. Louis, MO, USA) as described^30^, in α-MEM supplemented with 10% FBS, 2 mM L-glutamine, 100 U/ml penicillin, and 100 g/ml streptomycin (all from Invitrogen, Carlsbad, CA, USA) in a humidified atmosphere with 5% CO_2_ at 37℃. After 14 days, the cells were stained for TRAP with a commercial kit (Sigma-Aldrich, St. Louis, MO, USA) to determine the mature osteoclasts, and TRAP^+^ multinucleated cells with over three nuclei were identified as mature osteoclasts.

### Statistical analysis

All data are represented as the means ± standard deviation (SD). Intergroup analysis among Sham, GNX and MSCT groups was performed using two-way analysis of variance (ANOVA) followed by Bonferroni post hoc tests. Intragroup analysis for potential gender differences was performed using two-tailed Student’s *t* tests. The statistical analyses were conducted using the GraphPad Prism 5.1 software (San Diego, CA, USA). Values of *P* < 0.05 were considered to be statistically significant.

## Results

### Systemic MSCT rescues trabecular bone loss induced by gonadal steroid deficiency in both female and male mice

**Fig. 1:**
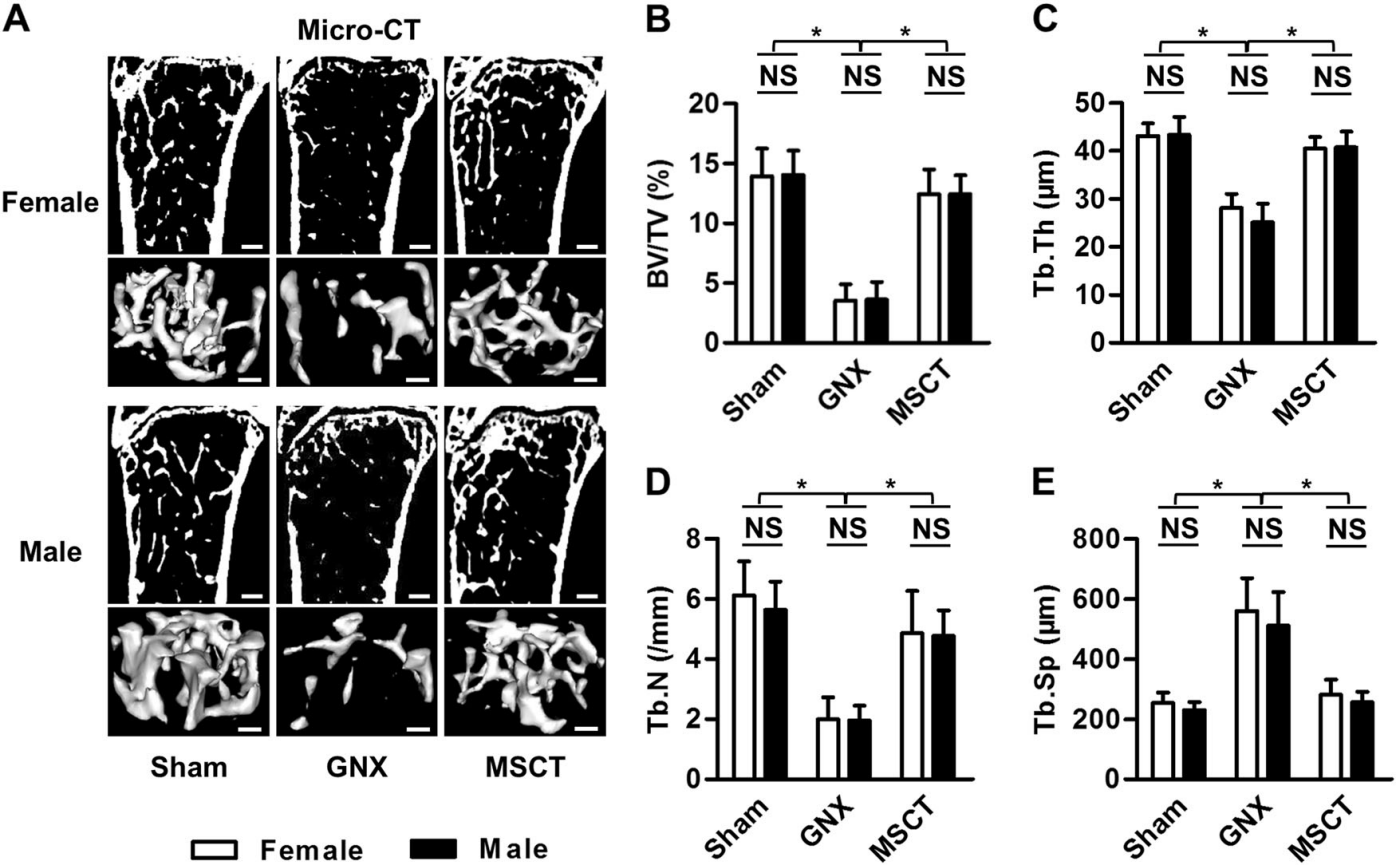
Trabecular bone mass and microarchitecture. **a** Micro-CT images of sections of the mouse distal femora and 3D reconstruction of the trabecular bone area. Bars: 300 μm (upper panels of each gender) and 100 μm (lower panels of each gender). GNX gonadectomy, MSCT mesenchymal stem cell transplantation in GNX mice. **b**–**e** Quantitative parameters of trabecular bone. BV/TV, bone volume over tissue volume; Tb.Th trabecular thickness, Tb.N trabecular number, Tb.Sp trabecular separation. *n* = 5 per group. The data represent the means ± SD. *P < 0.05; NS not significant (*P* > 0.05)

To examine the effects and the potential gender differences in the MSC therapy of gonadal steroid deficiency-induced osteoporosis, we first established GNX models in both female and male mice. As reported previously^20,31,32^ and confirmed in this study, mouse GNX induced comparable loss of trabecular bone mass in both genders (Fig. 1a, b) with similar impairments in the trabecular bone architecture (Fig. 1c–e). Then, we isolated mouse MSCs from bone marrow and verified their suitability for systemic cytotherapy (Supplementary Figure 2) according to published protocols^8,14^. We discovered that systemic MSCT restored the trabecular bone mass in both female and male GNX mice, as shown by representative micro-CT images and 3D reconstruction of the trabecular bone areas (Fig. 1a). Corresponding quantification of trabecular bone volume (Fig. 1b) and trabecular bone structure (Fig. 1c–e) confirmed the therapeutic efficacy of the MSC infusion in GNX-induced osteoporosis, and no difference was detected between genders. These results indicated that systemic MSCT rescues the trabecular bone loss induced by gonadal steroid deficiency in both female and male mice.

### MSC therapy restores cortical bone mass and bone quality in GNX mice of both genders

**Fig. 2:**
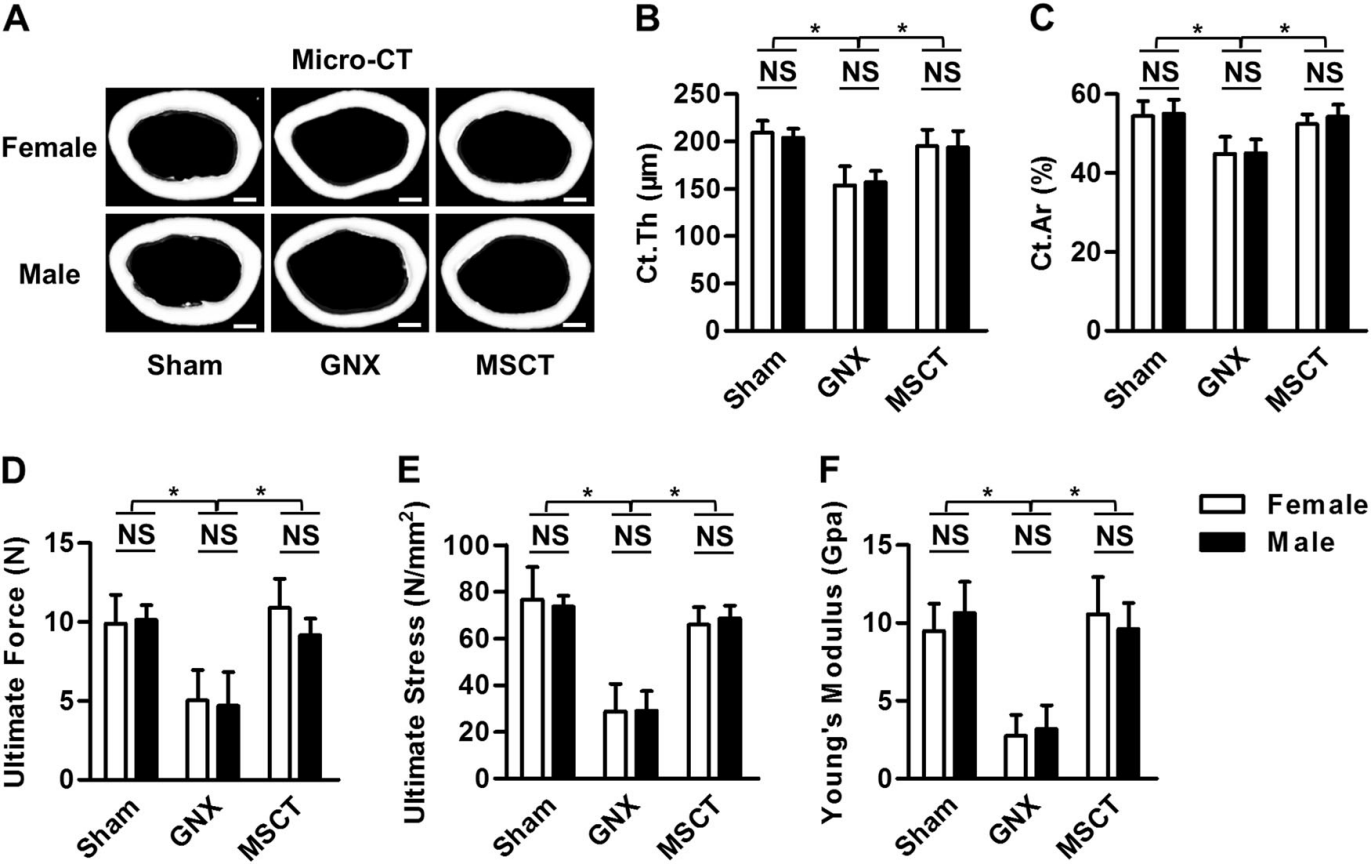
Cortical bone quantification and quality. **a** Micro-CT images of the cortical bone of mouse distal femora. Bars: 500 μm. GNX gonadectomy, MSCT mesenchymal stem cell transplantation in GNX mice. **b**, **c** Parameters of cortical bone. Ct.Th cortical thickness, Ct.Ar cortical area. **d**–**f** Results of the 3-point bending mechanical test. *n* = 5 per group. The data represent the means ± SD. *P < 0.05; NS not significant (*P* > 0.05)

Next, we investigated the effects of systemic MSCT on the cortical bone mass in GNX mice of both genders. The initial observations for this experiment showed that female and male mice developed paralleled cortical bone loss after GNX (Fig. 2a–c). As expected, MSC therapy remarkably ameliorated the cortical bone deficiency in both genders despite GNX (Fig. 2a), which led to uniformly restored cortical bone thickness (Fig. 2b) and bone area (Fig. 2c). Importantly, in the mechanical test, the MSC-treated GNX femora showed particular resistance to the exerted force, demonstrating significantly improved bone quality, and no difference was detected between genders (Fig. 2d–f). These data suggested that MSC therapy restores cortical bone mass and bone quality in both GNX female and male mice.

### Bone formation recovery is comparable between genders after MSC treatment of GNX-induced bone loss

**Fig. 3:**
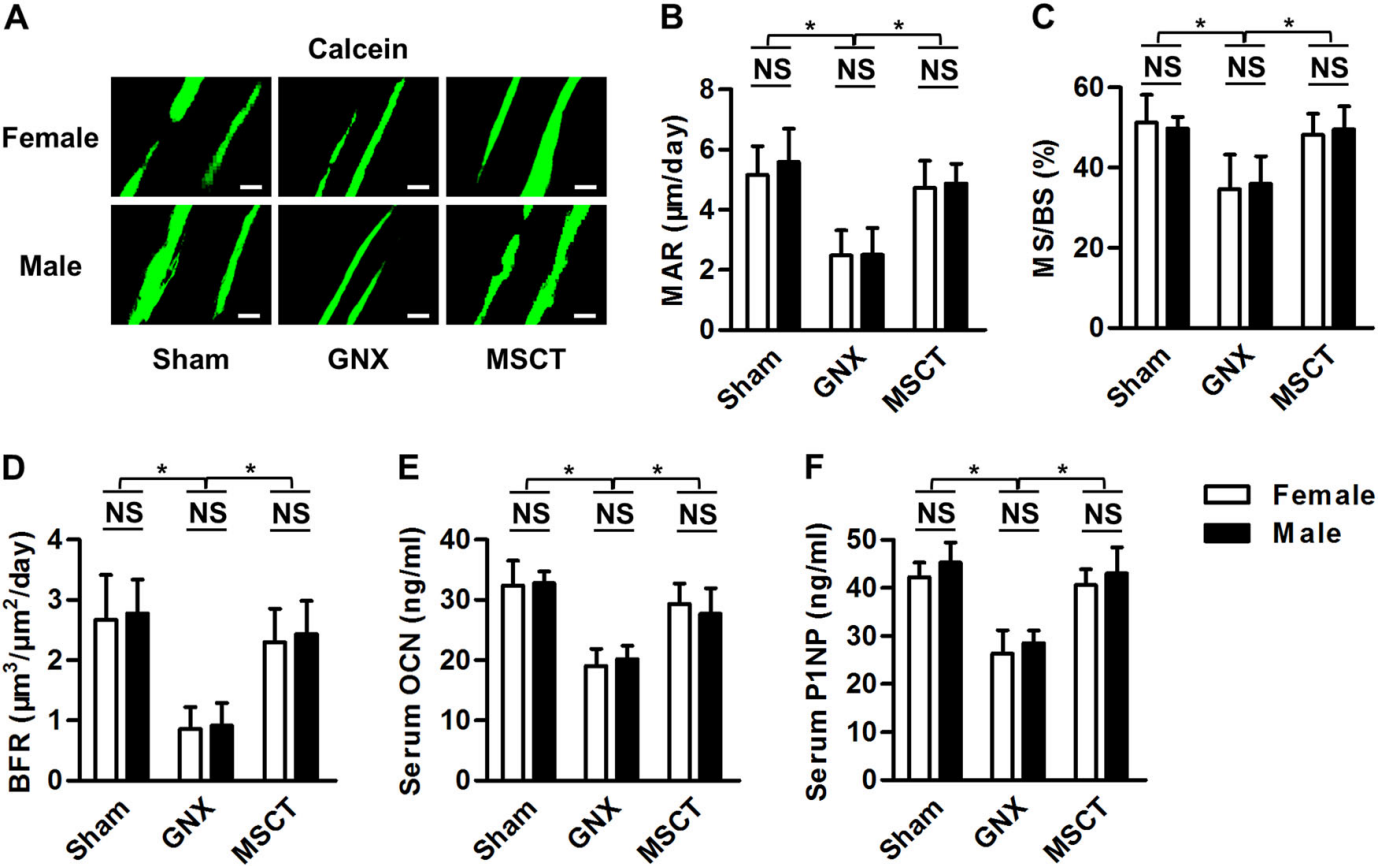
Bone formation examination. **a** Calcein double-labeling images of the mineralized surface of the mouse distal femora. Bars: 50 μm. GNX gonadectomy, MSCT mesenchymal stem cell transplantation in GNX mice. **b**–**d** Corresponding parameters of calcein labeling. MAR mineral apposition rate, MS/BS mineralized surface over bone surface, BFR bone formation rate. **e**, **f** Serological markers of bone formation. OCN osteocalcin, P1NP procollagen 1 N-terminal peptide. *n* = 5 per group. The data represent the means ± SD. *P < 0.05; NS, not significant (*P* > 0.05)

We next aimed to determine whether the paralleled efficacy of MSC in treating GNX-induced osteoporosis in female and male mice could be attributed to consistent recovery of bone remodeling balance, or that if any preferences existed between genders. For the osteoblastic bone formation, as demonstrated by calcein labeling, comparable inhibition could be detected after GNX in female and male mice, and systemic MSCT reversed this inhibition in both genders (Fig. 3a). Quantification of the corresponding parameters regarding mineral apposition (Fig. 3b), osteoblastic surface (Fig. 3c) and bone formation (Fig. 3d) confirmed that MSC therapy in GNX mice resulted in equally efficient recovery of osteoblastic bone formation in both genders. These effects were further verified systemically at the serological level using the bone formation markers OCN (Fig. 3e) and P1NP (Fig. 3f). The above findings suggested that bone formation recovery is comparable between genders in the MSC treatment of GNX-induced bone loss.

### Altered bone resorption in GNX osteoporosis is uniformly rectified by MSC infusion in female and male mice

**Fig. 4:**
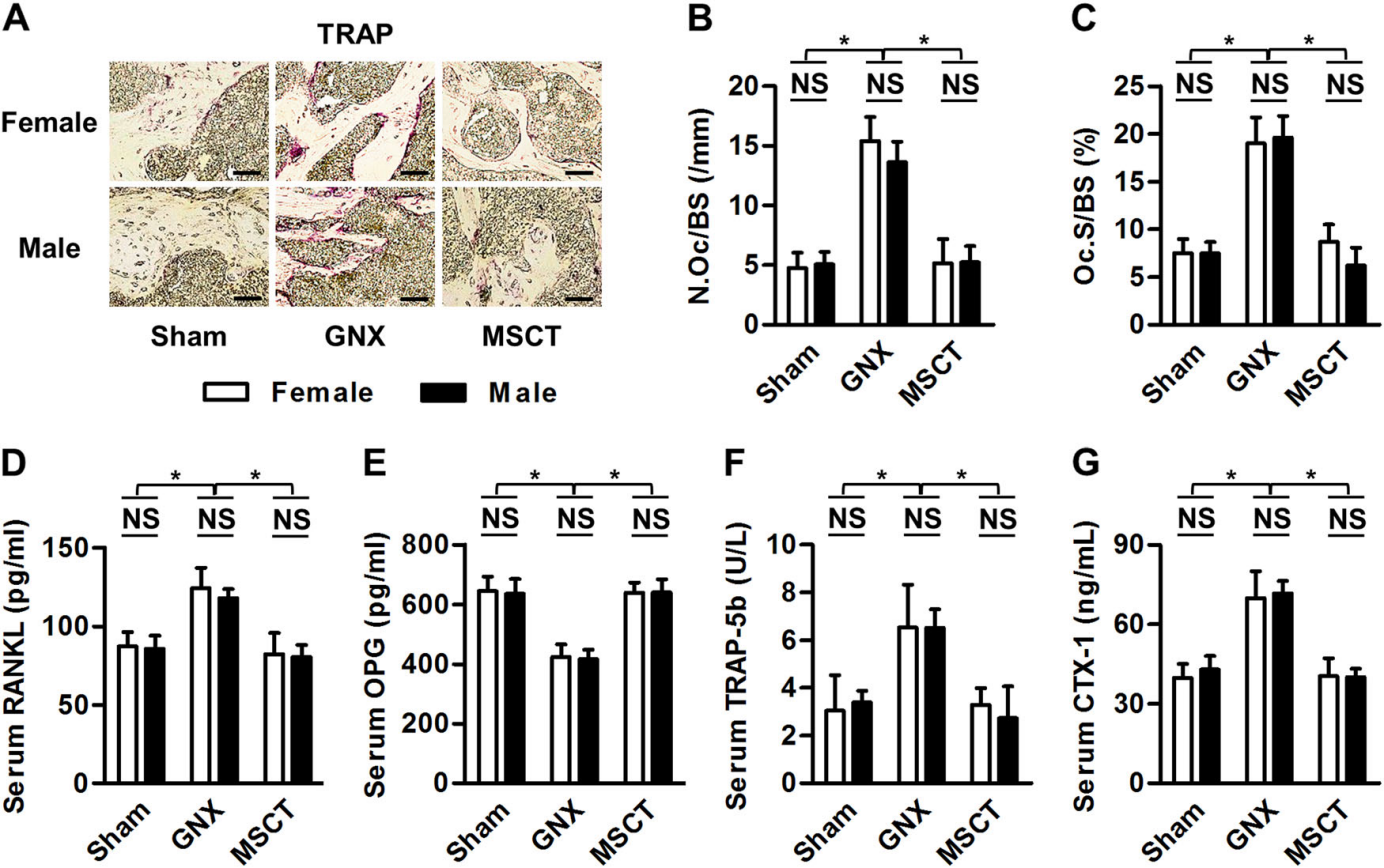
Bone resorption determination. **a** Tartrate-resistant acid phosphatase (TRAP) staining images of mouse distal femora. Bars: 50 μm. GNX gonadectomy, MSCT mesenchymal stem cell transplantation in GNX mice. **b**, **c** Corresponding parameters of TRAP staining. N.Oc/BS number of osteoclasts over bone surface, Oc.S/BS osteoclast surface over bone surface. **d**–**f** Serological markers of osteoclastogenesis. RANKL receptor activator of nuclear factor κB ligand, OPG osteoprotegerin. **g** The serological marker of bone resorption, CTX-1 cross-linked C-telopeptide of type 1 collagen. *n* = 5 per group. The data represent the means ± SD. *P < 0.05; NS, not significant (*P* > 0.05)

With regard to the osteoclastic bone resorption, we performed TRAP staining locally in the femora and detected consistent stimulation after GNX in both genders, and systemic MSCT effectively blocked the detrimental effects (Fig. 4a). Quantification of the osteoclastic parameters of number (Fig. 4b) and surface area (Fig. 4c) confirmed comparable therapeutic effects of infused MSCs on the localized alterations in bone resorption in both female and male GNX mice. To further examine the systemic changes as well as to clarify the underlying mechanisms for the osteoclastic restoration, we analyzed serological markers of osteoclastogenesis. The data demonstrated that in both female and male GNX mice, MSCT suppressed serological RANKL (Fig. 4d) but promoted OPG (Fig. 4e), leading to normalized levels of osteoclastic TRAP-5b (Fig. 4f) in the serum. Furthermore, the serological marker of bone resorption, CTX-1 (Fig. 4g), was also restored by the MSC therapy in both genders of mice that were subjected to GNX, which indicated systemic therapeutic effects. These findings suggested that the altered bone resorption in GNX osteoporosis is uniformly rectified by MSC infusion in female and male mice.

### Rapid engraftment but poor inhabitation of donor MSCs in GNX recipient bone marrow of each gender

**Fig. 5:**
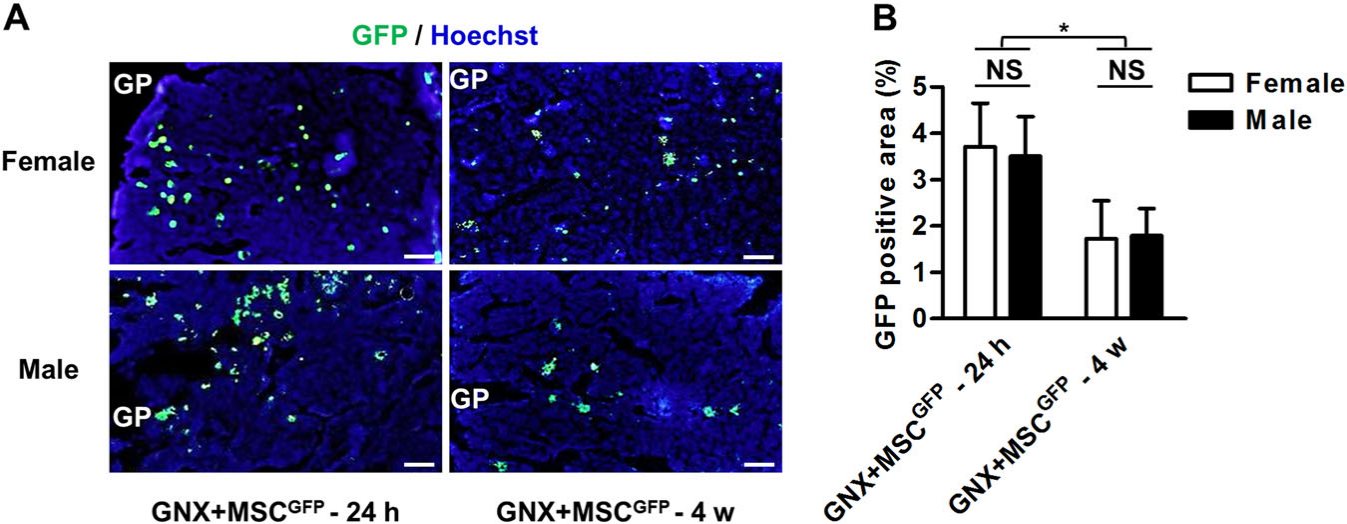
Bone marrow tracing of infused mesenchymal stem cells (MSCs). **a** Immunofluorescent staining of inhabited MSCs in recipient mouse bone marrow of distal femora after systemic transplantation. Donor MSCs were labeled with green fluorescent protein (MSC^GFP^). Bars: 100 μm. GP growth plate, GNX gonadectomy. **b** Corresponding quantification of engrafted GFP-labeled MSCs. *n* = 3 per group. The data represent the means ± SD. *P < 0.05; NS not significant (*P* > 0.05)

Next, we planned to dissect the mechanisms underlying the comparable therapeutic efficacy of MSCT in GNX osteoporosis of both genders. According to published papers, systemically transplanted MSCs rescue bone loss through either homing-based local effects^10,14,15^ or anti-inflammatory effects in the circulation^8,9,16^. Here, using donor MSCs derived from GFP transgenic mice, we were able to trace infused MSCs into recipient osteoporotic sites. We discovered that despite rapid homing of MSCs^GFP^ in GNX recipient bone marrow of both female and male mice, the engrafted donor MSCs and their putative lineage cells were not capable of inhabitation within the experimental period (Fig. 5a). Corresponding quantification confirmed that the engrafted MSCs^GFP^ accounted for less than 5% of the total GNX marrow area in the first 24 h, and the surviving population declined to only approximately 2% at the end of the observation period (Fig. 5b). Notably, female and male GNX mice demonstrated a parallel ability to accommodate donor MSCs in their bone marrow (Fig. 5b). These data indicated that homing-based local effects might not be the critical therapeutic mechanism underlying systemic MSCT in the treatment of GNX-induced osteoporosis, but the potential contribution (if any), was comparable between genders.

### Allogeneic MSCs efficiently suppresses T-cell and inflammatory responses in the treatment of female and male GNX-induced osteoporosis

**Fig. 6:**
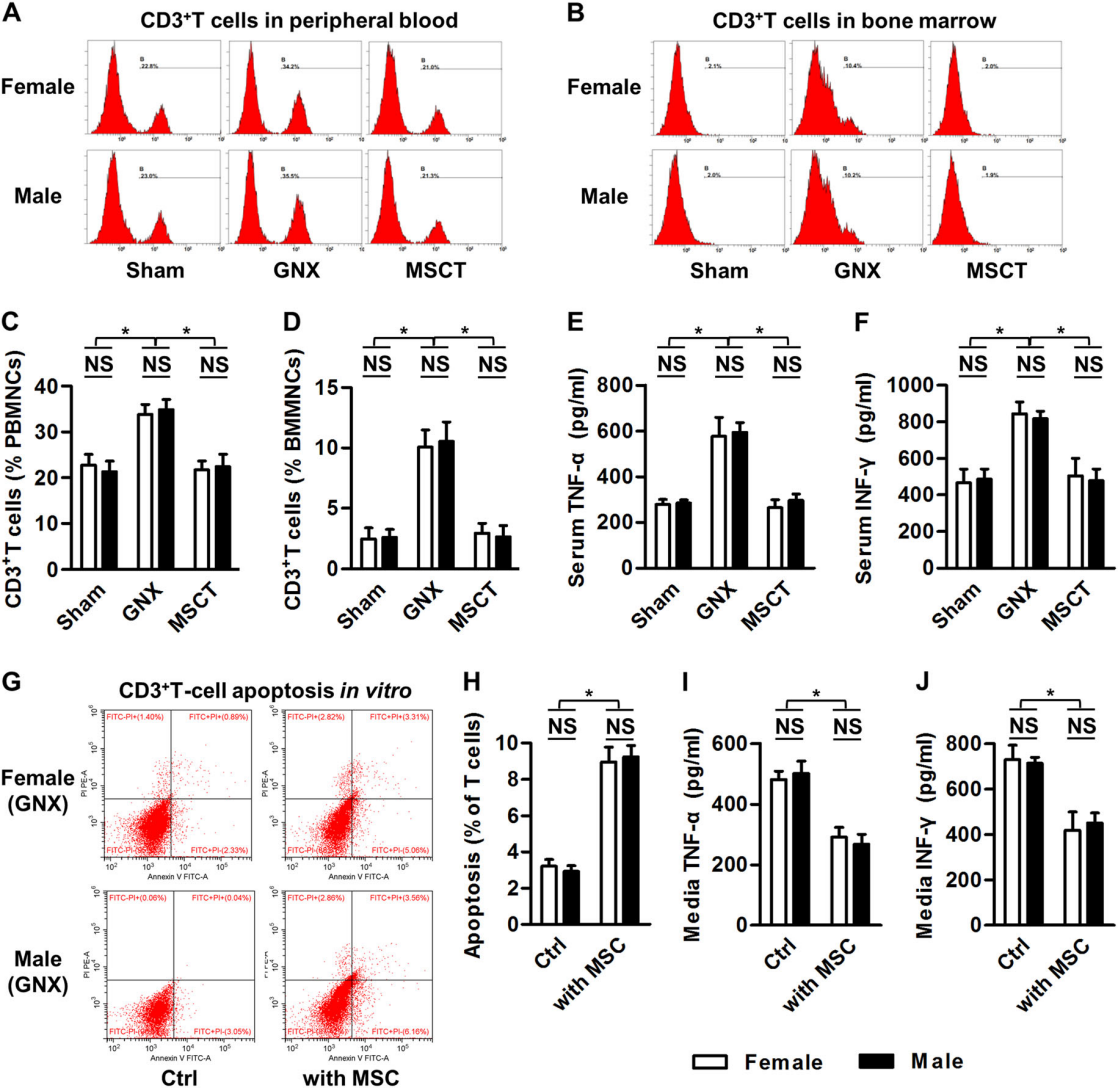
Total T-cell and inflammatory cytokine responses. **a**, **b** Flow cytometric analysis of systemic (circulation) and local (bone marrow) CD3^+^ T-cell populations. GNX, gonadectomy; MSCT, mesenchymal stem cell transplantation in GNX mice. (**c**, **d**) Corresponding quantification of total CD3^+^ T cells. PBMNCs peripheral blood mononuclear cells, BMMNCs bone marrow mononuclear cells. *n* = 3 per group. **e**, **f** Serological markers of inflammation. TNF-α tumor necrosis factor-alpha, IFN-γ interferon-gamma, *n* = 5 per group. **g**, **h** Flow cytometric analysis of the apoptotic rate of CD3^+^ T cells from GNX mice in co-culture with or without mesenchymal stem cells (MSCs). **i**, **j** Inflammatory cytokine concentrations in the co-culture media. *n* = 3 per group. The data represent the means ± SD. *P < 0.05; NS not significant (*P* > 0.05)

The above findings prompted us to investigate whether microenvironmental effects were the primary therapeutic mechanism in the reversal of GNX-induced osteoporosis by MSC in both genders. As stated before, estrogen deficiency leads to expansion of a T-cell population with enhanced production of key inflammatory cytokines TNFα and IFN-γ, which results in bone loss^33–35^. Accordingly, through anti-inflammatory effects that suppress the T-cell population, systemic MSCT is sufficient to prevent or treat OVX-induced osteoporosis^8,9^. In this study, we further verified that similar to the mouse OVX model, ORX in mice also triggered a substantial increase in the T-cell population (Fig. 6a–d), which was associated with the onset of systemic inflammation (Fig. 6e, f). Importantly, systemic MSCT remarkably inhibited the expansion of the CD3^+^ T cells in both the circulation (Fig. 6a) and local bone marrow (Fig. 6b) of GNX mice, and no difference was detected between genders (Fig. 6c, d). This suppression of the total T-cell population led to a suppression of the increases in inflammatory indicators in both genders, as indicated by the reduced serum levels of TNFα and IFN-γ in GNX mice that received MSC therapy (Fig. 6e, f).

To prove directly the immunosuppressive and anti-inflammatory effects of allogeneic MSCs, we used an in vitro T-cell—MSC co-culture system^8,17^. We found that after co-culture with allogeneic MSCs for only 6 h, T cells from GNX mice of both genders underwent a remarkable stimulation of apoptosis, which could account for the reduced T-cell population in vivo (Fig. 6g, h). Furthermore, this induced T-cell apoptosis resulted in lower concentrations of TNFα and IFN-γ in the co-culture media, suggesting an anti-inflammatory effect of MSCs (Fig. 6i, j). Interestingly, T cells from the Sham mice showed similar responses to allogeneic MSCs, which indicated strong immunosuppression by the MSCT (Supplementary Figure 3). Taken together, these results emphasize that MSCT efficiently suppresses T-cell and inflammatory responses, which critically contribute to the reversal of both female and male GNX-induced osteoporosis.

### Systemic delivery of MSCs restored functional disorders of endogenous MSCs

**Fig. 7:**
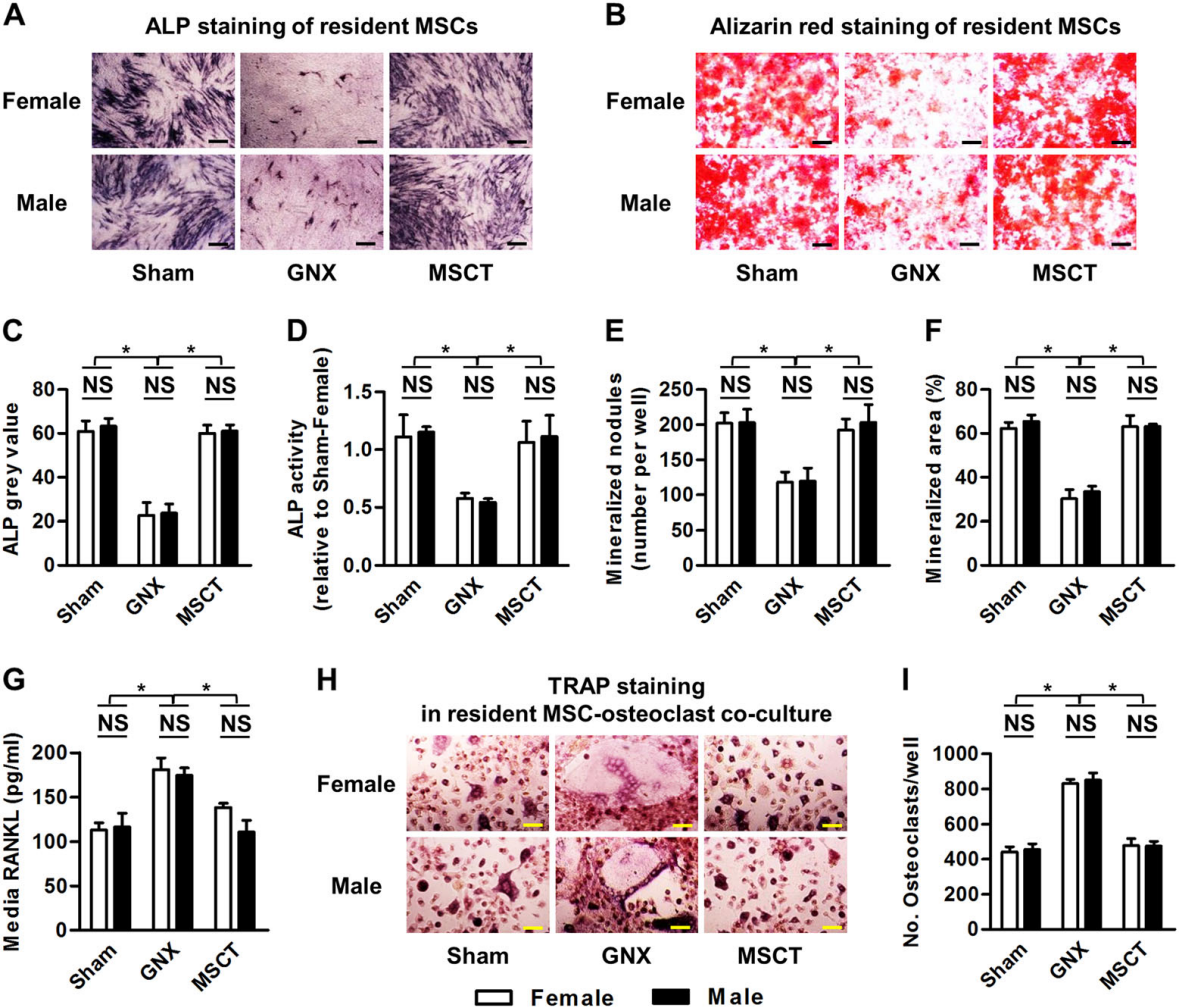
Osteogenesis of resident mesenchymal stem cells (MSCs) and their capability to induce osteoclastogenesis. **a**–**f**) Alkaline phosphatase (ALP) staining (**a**), alizarin red staining (**b**) and the quantifications in osteogenesis of resident MSCs (**c**–**f**). GNX gonadectomy, MSCT mesenchymal stem cell transplantation in GNX mice. **g** The concentration of the key osteoclastogenesis-factor, Receptor or Activator of NF-kappaB Ligand (RANKL), in MSC media. **h**, **i** Tartrate-resistant acid phosphatase (TRAP) staining for osteoclastogenesis in co-culture of MSCs and osteoclasts. *n* = 3 per group. The data represent the means ± SD. *P < 0.05; NS not significant (*P* > 0.05)

To further understand the biological basis for the reversal of GNX-induced osteoporosis by MSCT through downstream microenvironmental modulation, we analyzed the behaviors of the resident MSCs, which are gradually recognized as critical contributors to bone homeostasis by osteogenic differentiation and regulation of osteoclastogenesis, among other effects^4,5^. We discovered that endogenous MSCs from GNX mice of both genders showed dramatic declines in osteogenesis, as demonstrated by analyses of ALP activity and mineralization capability (Fig. 7a–f). Importantly, in both male and female GNX mice, the MSCT significantly reversed the osteogenic defects in resident MSCs. Furthermore, to assess osteoclastogenesis by resident MSCs, we analyzed the RANKL levels in the media and demonstrated that MSCT restored the ability of endogenous MSCs from GNX mice of both genders to secrete RANKL (Fig. 7g). These effects further contributed to alterations of osteoclastogenesis when osteoclasts were co-cultured with resident MSCs (Fig. 7h, i). Collectively, these data indicated that systemic delivery of allogeneic MSCs reversed the functional disorders of endogenous MSCs in the treatment of GNX-induced osteoporosis in both genders.

## Discussion

Osteoporosis develops with high prevalence in hypogonadism of both women and men, but therapeutic approaches to treatment have not yet been established^1–3^. In recent years, an immense potential for MSCs for cytotherapy of osteoporosis has emerged. However, the effects, mechanisms and the potential gender differences in the MSC therapy of gonadal steroid deficiency-induced bone loss are not fully understood^8–12^. Here, we determined that systemic MSCT rescued both trabecular and cortical bone loss as well as restoring the bone quality in female and male GNX mice, and no difference was detected between genders. Furthermore, MSCT demonstrated equal efficiency in rectifying the bone remodeling balance in GNX mice of both genders, as demonstrated by the comparable recovery of bone formation and paralleled normalization of bone resorption. Mechanistically, we demonstrated rapid engraftment but poor inhabitation of donor MSCs in the bone marrow of GNX recipient mice of each gender, which indicated that homing-based local effects might not be the critical therapeutic mechanism. Alternatively, systemic delivery of MSCs uniformly reduced CD3^+^T-cell population and suppressed the inflammatory responses in the treatment of female and male GNX-induced osteoporosis. Eventually, resident MSCs in GNX mice were functionally restored upon the microenvironmental modulation induced by allogeneic MSCs. Taken together, these data revealed recipient sexual monomorphic responses to MSC therapy in gonadal steroid deficiency-induced osteoporosis via immunosuppression/anti-inflammation and endogenous stem cell recovery.

Common and differential gender influences on bone metabolism have been revealed^13,36^. It has been documented that gender differences may be present in human bone development and in peak bone mass, although bone mass accrual before and during puberty can be similar in women and men, Similar age-specific risks of fractures are also observed^37,38^. In osteoporosis with hypogonadism, despite original reports claiming different patterns of bone loss^39,40^, recent animal studies that have used OVX and ORX identified consistent osteoporotic phenotypes with similar bone remodeling alterations^20,41^. Gender variability also exists in skeletal responses to pharmacological therapeutics, which has demonstrated either sexual monomorphic or dimorphic restorations after GNX in rodents^31,42^. The current knowledge indicates that sex steroids and their receptors serve as the putative key contributing factors. Estrogen is recognized to be one of the most important hormones for bone development and maintenance. This hormone controls the bone remodeling process not only via direct modulation of osteoclastic as well as osteoblastic cell genesis and function but also indirectly through regulation of the immune system to adjust cytokine production^43–45^. Accordingly, estrogen deficiency in both women (postmenopause) and female rodents (OVX) predisposes to osteoporosis by causing an uncoupling between bone resorption and bone formation, which is at least partially triggered by the secondary onset of systemic inflammation^18,46,47^. Androgen, on the other hand, plays an important role in the maintenance of the male skeleton, although it remains unclear if this is a direct effect of testosterone or a result of estradiol transformation through aromatization^48,49^. Regardless of the hormonal participation, hypogonadism in men (aging, idiopathic hypogonadotropic hypogonadism and after castration or androgen-deprivation therapy) and male mice (ORX) results in significant bone loss, which is attributed to bone remodeling alterations^50–54^. Together with previous studies^20,41^, the present study confirmed that gonadal steroid deficiency actually induces similar osteoporotic phenotypes in mice, including loss of trabecular and cortical bone mass, impaired bone quality, stimulated bone resorption, repressed bone formation and increased marrow adiposity. Furthermore, we demonstrated that GNX in both genders provokes an inflammatory profile due to the consistent T-cell population expansion, which is the critical pathological factor that leads to osteoclastic activation and a differentiation switch in endogenous MSCs from osteoblastogenesis toward adipogenesis^20,41^. Therefore, it can be concluded that deficiency of differential gonadal hormones induce common skeletal pathogenesis in rodents, which should be verified in clinics.

MSCs prevent or treat osteoporosis through mechanisms basically categorized into two aspects: local and systemic, or direct and indirect^4^. It has been reported that via intra-bone marrow injection or genetically manipulated enforced homing after systemic infusion, MSCT alleviate the murine bone loss induced by OVX and related to aging, but these methodologies are either traumatic or risky^10,55,56^. Furthermore, despite the possibility that natural MSCs can also engraft to local osteoporotic sites and exert anabolic effects by direct differentiation and/or the secretion of factors trophic to the recipient bone cells, the donor MSC inhabitation only conditionally occurs in an immunosuppressive status under glucocorticoids^14^. In the present study, together with our previous documentation^8^, we confirmed that in the inflammatory microenvironments of GNX female and male mice and in diabetes, the weak homing and inhabitation of donor MSCs to recipient bone marrow poorly supports a functional integration. Alternatively anti-inflammatory effects in the circulation may lead to general bone improvements of both anabolic and anti-catabolic effects on the basis that impairment of anti-inflammatory capability resulted in the loss of osteoporotic therapeutic efficacy^8,9^. At the cellular level, the anti-inflammatory function of MSCs is based on their ability to induce T-cell apoptosis, which at the molecular level is attributed to Fas-Fas ligand (Fasl)-mediated cell-cell interaction and the secondary normalization of T-cell subsets^8,9,17^, although paracrine mechanisms of cytokine and exosomal release might also contribute to osteoporotic cytotherapy^12,57,58^. Notably, although several studies have reported potential gender differences in the MSC treatment of certain diseases that were related to the expressed hormonal receptors^59,60^, these studies focused only on the sexes of the donors rather than those of the recipients. In the present study, this mechanism was ruled out by the application of the mixed MSCs from both genders in the GNX model. Therefore, it can be proposed that systemic MSCT reverses the osteoporosis induced by gonadal steroid deficiency through anti-inflammatory effects without differentiating between the female and male recipients.

Resident MSCs in bone marrow account for the major progenitor cell population that putatively contributes to bone homeostasis and diseases^4^. It has gradually been acknowledged that adult MSCs reside in a niche enriched with neurovascular components, where they are actively modulated by systemic or local microenvironmental factors such as hormones, cytokines and metabolites^5^. Previously, it has been documented that under estrogen deficiency-induced osteoporosis, elevated inflammatory cytokines impair osteogenesis of MSCs through multiple signaling and posttranscriptional factors^18,19^, whereas the loss of the sex hormone enhances the MSC ability to induce osteoclastogenesis^21^. These functional alterations of endogenous MSCs have emerged as critical mediators of the pathogenesis of bone loss under the estrogen-deficient status. In this study, we further showed that allogeneic MSCT remarkably normalized the disordered functions of resident MSCs in GNX-induced osteoporosis, which suggested that the recovery of the endogenous MSC after immunomodulation is significant for this treatment of bone loss.

In conclusion, systemic MSCT not only reverses trabecular bone loss but also restores cortical bone mass and bone quality in GNX mice, and there were no therapeutic differences between the genders. These effects are attributed to the equal efficacy of MSC therapy in rectifying the bone remodeling balance in GNX mice of both genders. Mechanistically, the uniformly reduced total T-cell population and suppressed inflammation, rather than homing-based local effects, accounted for the functional recovery of the endogenous stem cells as the critical mechanism for the MSCT-induced treatment of female and male GNX osteoporosis. In summary, our data uncovered recipient sexual monomorphic responses to MSC therapy in gonadal steroid deficiency-induced osteoporosis via immunosuppression/anti-inflammation and resident stem cell recovery, which shed light on the gender-independent clinical utility of this therapy.

## Supplementary Information

Supplemenatry Information
