# Supplementary material for: Gender-independent efficacy of mesenchymal stem cell therapy in sex hormone-deficient bone loss via immunosuppression and resident stem cell recovery
Source: Exp Mol Med. 2018 Dec 17;50(12):166. doi: 10.1038/s12276-018-0192-0 (PMC6297134; doi:10.1038/s12276-018-0192-0)
Supplement: Supplementary file 1 — Supplemenatry Information [file 12276_2018_192_MOESM1_ESM.docx]

***Supplementary Information***

**Gender-independent efficacy of mesenchymal stem cell therapy in sex hormone-deficient bone loss via immunosuppression and resident stem cell recovery**

**RUNNING HEAD:** Gender-independence of MSC in treating GNX-induced osteoporosis

Bing-Dong Sui,^1, 2, †^ Ji Chen,^1, 2, †^ Xin-Yi Zhang,^1, 2, †^ Tao He,^1, 2^ Pan Zhao,^1, 3^ Chen-Xi Zheng,^1, 2^ Meng Li, ^1^ Cheng-Hu Hu^1, 3, *^ and Yan Jin^1, 2, *^

^1^ State Key Laboratory of Military Stomatology, Center for Tissue Engineering, Fourth Military Medical University, Xi’an, Shaanxi, 710032, China.

^2^ Research and Development Center for Tissue Engineering, Fourth Military Medical University, Xi’an, Shaanxi 710032, China.

^3^ Xi’an Institute of Tissue Engineering and Regenerative Medicine, Xi’an, Shaanxi, 710032, China.

^†^ Bing-Dong Sui, Ji Chen and Xin-Yi Zhang contributed equally to this work.

**^*^CORRESPONDENCE**

**Prof. Yan Jin** and **Dr. Cheng-Hu Hu**, State Key Laboratory of Military Stomatology, Center for Tissue Engineering, Fourth Military Medical University, No. 145 West Changle Road, Xi’an, Shaanxi 710032, China. *E-mail:* yanjin@fmmu.edu.cn (Prof. Yan Jin), lshchoo@qq.com (Dr. Cheng-Hu Hu); *Tel:* +86-029-84776472; *Fax:* +86-029-83218039.

**
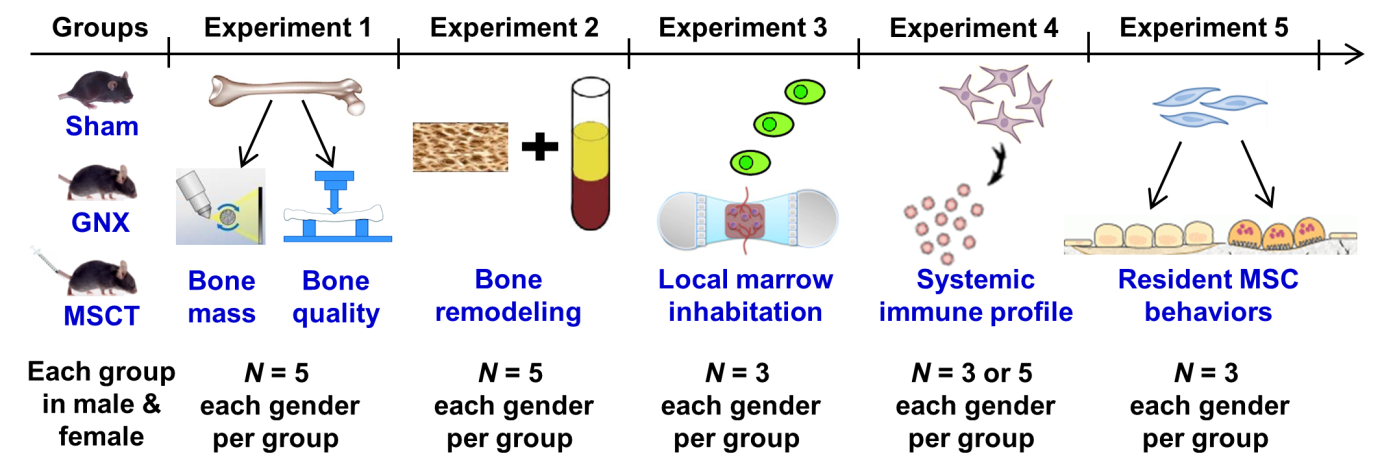
**

**Supplementary Figure 1.** Flow chart demonstrating experimental design of this study. Mice were divided into 3 groups as Sham, gonadectomy (GNX), and mesenchymal stem cell transplantation (MSCT) in GNX mice. Each of the 3 groups were equally composed of male and female individuals. Experiment 1-5 were designed based on each specific purpose from bone mass and quality examination to mechanistic investigations at the tissue and cell levels in local and systemic microenvironment.

**
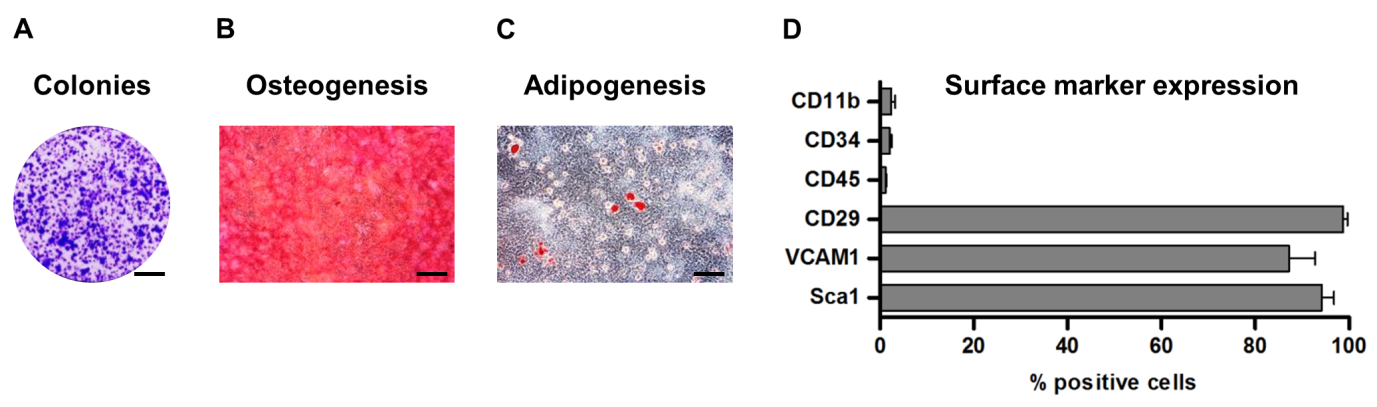
**

**Supplementary Figure 2.** Identification of MSCs. (**A**) Colony formation of adherent primary MSCs derived from mouse bone marrow. Colonies were stained by crystal violet. Bar: 1 cm. (**B**) Mineralization of MSCs in osteogenic differentiation, demonstrated by alizarin red staining. Bar: 100 μm. (**C**) Lipid droplet formation of MSCs in adipogenic differentiation, demonstrated by oil red O staining. Bar: 100 μm. (**D**) Surface marker analysis by flow cytometry. VCAM1, vascular cell adhesion molecule 1/CD106. Sca1, stem cell antigen 1. *N* = 3 per marker. Data represent mean ± SD.


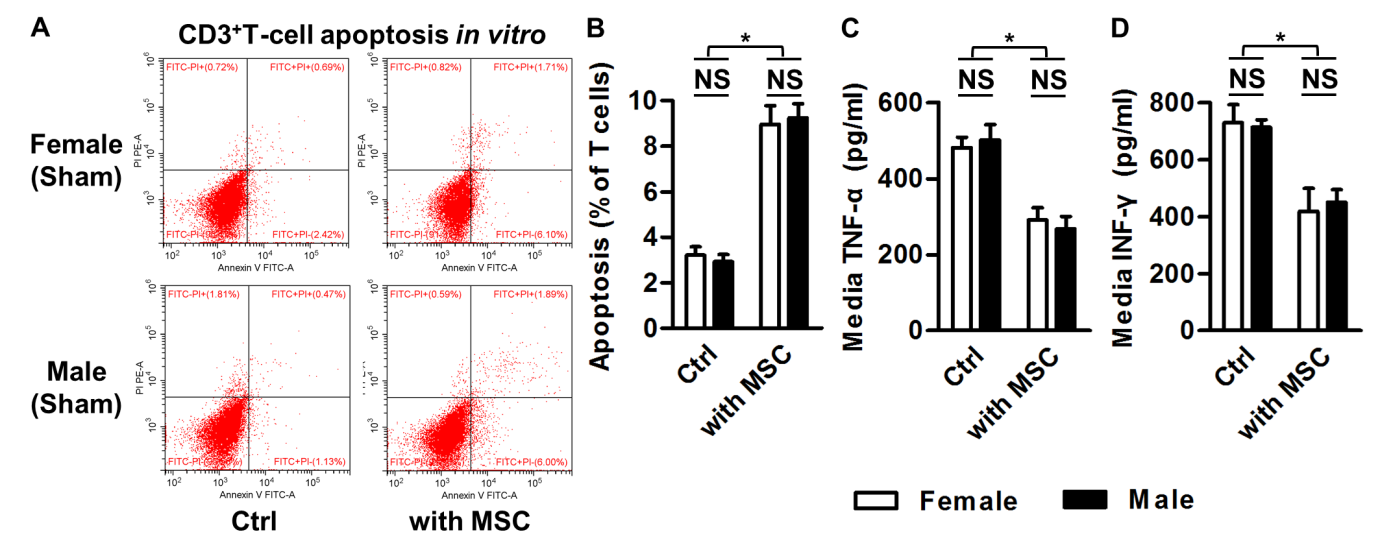
**Supplementary Figure 3.** T-cell and inflammatory cytokine responses in Sham model. (**A, B**) Flow cytometric analysis of apoptotic rate of CD3^+^ T cells from Sham mice in co-culture with or without mesenchymal stem cells (MSCs). (**C, D**) Inflammatory cytokine concentrations in the co-culture media. *N* = 3 per group. Data represent mean ± SD. *, *P* < 0.05; NS, not significant (*P* > 0.05).
